# Intrusion Detection and Prevention in CoAP Wireless Sensor Networks Using Anomaly Detection

**DOI:** 10.3390/s18082445

**Published:** 2018-07-27

**Authors:** Jorge Granjal, João M. Silva, Nuno Lourenço

**Affiliations:** Centre for Informatics and Systems, Department of Informatics Engineering, University of Coimbra Polo 2, 3030-290 Coimbra, Portugal; jcbsilva@student.dei.uc.pt (J.M.S.); naml@dei.uc.pt (N.L.)

**Keywords:** intrusion detection, anomaly detection, 6LoWPAN, CoAP, internet-integrated sensor networks

## Abstract

It is well recognized that security will play a major role in enabling most of the applications envisioned for the Internet of Things (IoT). We must also note that most of such applications will employ sensing and actuating devices integrated with the Internet communications infrastructure and, from the minute such devices start to support end-to-end communications with external (Internet) hosts, they will be exposed to all kinds of threats and attacks. With this in mind, we propose an IDS framework for the detection and prevention of attacks in the context of Internet-integrated CoAP communication environments and, in the context of this framework, we implement and experimentally evaluate the effectiveness of anomaly-based intrusion detection, with the goal of detecting Denial of Service (DoS) attacks and attacks against the 6LoWPAN and CoAP communication protocols. From the results obtained in our experimental evaluation we observe that the proposed approach may viably protect devices against the considered attacks. We are able to achieve an accuracy of 93% considering the multi-class problem, thus when the pattern of specific intrusions is known. Considering the binary class problem, which allows us to recognize compromised devices, and though a lower accuracy of 92% is observed, a recall and an F_Measure of 98% were achieved. As far as our knowledge goes, ours is the first proposal targeting the usage of anomaly detection and prevention approaches to deal with application-layer and DoS attacks in 6LoWPAN and CoAP communication environments.

## 1. Introduction

It is a well known fact that most of the applications envisioned for the IoT will be supported (at least partially) by sensing and actuating devices which are constrained in terms of the available energy, memory and computational performance. The integration of such devices with the Internet communications infrastructure will enable end-to-end communications with other devices, anywhere on the Internet, but also new avenues for attacks from such less resource-constrained hosts. The aforementioned integration scenario is becoming a reality, thanks to the design and adoption of a standardized communications stack being designed for the IoT [1]. This stack is enabled by protocols such as 6LoWPAN (6LoWPAN adaptation layer) [2], Routing Protocol Layer (RPL) [3] and CoAP Constrained Application Protocol (COAP) [4]. We verify that there is currently a lack of proposals in the literature focusing on the specificities of detecting and dealing with attacks against the security and stability of 6LoWPAN and CoAP devices and communication environments. With this motivation in mind, in this article we propose an anomaly-based intrusion detection and prevention framework for Internet-integrated CoAP sensor networks, in the context which we implement and experimentally evaluate the effectiveness of complementary anomaly-based intrusion detection techniques in dealing with DoS attacks and attacks against the operations of the 6LoWPAN and CoAP protocols.

In our experimental methodology, we start by programming CoAP intrusion (attack) scenarios using the Contiki operating system [5] via the IoT-LAB [6] platform. The traffic generated during the experiments is used to apply feature extraction and complementary machine-learning approaches, validating the models built on top of the classification algorithms employed. We thus analyze the traffic patterns employing 6LoWPAN and CoAP communications, with the goal of training a machine learning algorithm to detect abnormal or suspicious communications. Our article proceeds as follows. In Section 2 we identify the challenges of intrusion detection and prevention in the context of IoT communication environments, and observe the current shortage of solutions designed for Internet-integrated 6LoWPAN and CoAP communication environments. In Section 3 we present the proposed framework for intrusion detection and prevention, and the techniques employed in our work to detect anomalies. In Section 4 we analyze the results obtained from the experimental evaluation of the proposed approach, and in Section 5 we conclude the article and discuss future research work in this area.

## 2. Security and Intrusion Detection in the IoT

We begin by analyzing security in the context of Internet-integrated sensor networks, more precisely how intrusion detection may be applied to IoT CoAP communication environments, as is our goal.

### 2.1. Security in the Context of Internet-Integrated Sensor Networks

A standardized communications stack is currently being formed with the purpose of enabling IP communications with constrained sensing and actuating devices [1]. In this context, protocols such as 6LoWPAN [2], RPL [3] and CoAP [4] have been designed to run over IEEE.802.15.4 [7] physical (PHY) and MAC layer communications, and other technologies are also being adopted by the 6LoWPAN adaptation layer, as is the case of Bluetooth Low Energy (BLE) [8]. IEEE 802.15.4 is inherently a link-layer communications technology, and as such only offers security to hop-by-hop communications. As this stack enables end-to-end communications (at the network and higher layers) between Internet-integrated constrained wireless sensing devices and other Internet entities, attacks against such devices may be diverse and take place at all layers of the stack. For example, in IEEE 802.15.4, confirmation (ACK) packets are not encrypted, and the knowledge of the numbering of the packet to be confirmed may be sufficient to perform a replay attack. As for the 6LoWPAN [2] adaptation layer, it was designed without security mechanisms, although proposals exist in the literature in this context [9]. Regarding routing operations performed over 6LoWPAN, Routing Protocol Layer (RPL) [3] defines a framework capable of transporting secure versions of the routing messages, as well as a set of mandatory cryptographic algorithms to be supported by sensing devices. Despite such functionalities, we find that RPL is vulnerable to attacks such as rank attacks, local repair attacks and resource depleting attacks [10].

In our work we consider that a particular focus on the Constrained Application Protocol (CoAP) [11] is of importance, given that it promises to play a major rule in enabling the IoT. CoAP was designed with the purpose of extending the REpresentational State Transfer (REST) architecture of the web to encompass sensors and actuators. The CoAP protocol inherits the same Create, Read, Update, Delete (CRUD) operations as ReST, the same applying to the error codes, and also shares some URL similarities. Regarding security, other than introducing security at the application-layer itself, CoAP delegates its support to the Datagram Transport Layer Security (DTLS) protocol at the transport-layer, with the goal of transparently securing communications between devices. Devices may authenticate using DTLS with pre-shared keys, public-keys or X.509 digital certificates. It is important to note that, despite the fact that DTLS offers transparent security for end-to-end communications between devices, it does not protect such devices against a number of external and internal attacks. Among such attacks we may find cache manipulations, which make the devices susceptible to man in the middle attacks, Distributed Denial of Service (DDoS) amplified attacks, spoofing attacks and cross-layer attacks, which may allow to bypass firewalls. On the other hand, internal attackers may try to subvert the normal operations of the CoAP protocol, as well as its semantic rules. Therefore, end-to-end security mechanisms may be complemented by appropriate detection and prevention approaches deployed in the context of the Internet-integrated WSN, as is our motivation in this work.

### 2.2. Intrusion Detection Approaches on the Internet

Before delving into the problem of detecting attacks in IoT CoAP communication environments, we find it useful to observe how intrusion detection is approached in the current Internet communications infrastructure. According to [12], IDS solutions currently employed in the Internet are classified in one of three major categories: signature-based detection (SD), anomaly-based detection (AD) and stateful protocol analysis (SPA). SD intrusion detection [13] (also known as knowledge-based detection or misuse detection) employs patterns or strings (signatures) of known attacks. As for AD intrusion detection [14] (also known as behavior-based intrusion detection), an anomaly is characterized as a deviation from the normal (expected) behavior of the network, with such behavior being characterized via a profile built from monitoring regular activities of the network. Profiles can be either static or dynamic, and are defined by a set of attributes which, in the case of a system, may include the frequency of key strokes by a user in a given system, the number of files accessed on a given time period or the number of incorrect attempts on the login screen, among others. Finally, with SPA intrusion detection [15] (also known as specification-based systems), suspicious activities are detected using profiles defined for specific protocols or applications.

It is also useful to consider how the technologies related with intrusion detection are employed in practical systems and, in this case, we find host-based IDS (HIDS), network-based IDS (NIDS) and wireless-based IDS (WIDS) [12]. HIDS implementations are mostly focused on monitoring and detecting suspicious activities in hosts, and usually are capable of monitoring all or parts of the dynamic behavior and state of the computer system, when compared with a baseline built during its initial setup. As for NIDS, such systems monitor a network or systems for malicious activities or policy violations, by analyzing the network traffic captured at strategic locations of the communications infrastructure. WIDS are similar to NIDS, while in this case being more focused on the monitoring of wireless communications.

### 2.3. Intrusion Detection on the IoT

As previously discussed, we consider that attacks against the security and stability of Internet-integrated constrained sensing and actuating devices may take place at the various layers of the communications stack, thus from the IEEE 802.15.4 [7] link layer up to the application layer using CoAP [4]. For example, we may find jamming and collisions attacks at the link layer, while at the application layer flooding and overwhelming of CoAP communications are two examples of attacks against this communications protocol [10]. On [16], jamming, cloning of things and eavesdropping are identified as possible DoS attacks against IoT communication environments, either at the routing or application layers. In [17] a solution is described to prevent DoS attacks against sensing devices originated at the Internet, by employing more capable devices and edge routers in the support of security-related operations. In this proposal, the routers also authorize the forwarding of communications between the WSN and the Internet domains, for the devices that pass a set of predefined conditions. In [16], a system is proposed to secure IoT devices via encryption and policy enforcement mechanisms employing Suricata [18] as an SD IDS. Authors in [19] propose SVELTE, an hybrid IDS for the detection of attacks against routing using RPL, which merges the characteristics of SD and AD intrusion detection. This proposal is evaluated through simulation with good results in dealing with sinkhole attacks.

In [20] the authors propose REATO, a rule-based solution for actively and dynamically detecting and dealing with DoS attacks in the context of a cross-domain IoT middleware. This system is designed to react to various situations in order to block undesired communications, and the proposal is implemented and evaluated experimentally, and found to be viable in defending from DoS attacks in an IoT scenario. In [21] a hybrid IDS (RIDES) is proposed with the goal of dealing with Ping of Death (PoD) attacks. RIDES is based on two main components, the signature code generator, which uses bloom filters for storing signature codes for Snort [22], and the network anomaly detector, which employs cumulative sum control charts for detecting abnormal network activity. The authors argue that RIDES can reduce energy consumption by 8 μJ using signature codes, and it is shown that the true positive ratio increases with the time interval.

We also find [23,24] as proposals of anomaly-based detection systems. The two proposals focus on preventing botnet attacks and on the usage of TCP communications, thus not being compliant with the usage of 6LoWPAN and CoAP, which currently run over UDP. We may find also other proposals in the literature with different methodologies, such as [25] for wormhole detection, Reference [26] based on Deep Packet Inspection (DPI) and [27]. In conclusion, and regarding the previously discussed proposals, we note that most of the implemented pure AD solutions are not extensively described in the literature. We also note that none of the existing proposals target the detection and handling of attacks at the CoAP application-layer, as per our motivation in this work.

## 3. Anomaly-Based Intrusion Detection in Internet-Integrated CoAP WSN

We now present the system architecture in the context of which we implement intrusion detection, in particular for the detection of DoS attacks and attacks at the application-layer against the CoAP Protocol.

### 3.1. Considered Approach

The approach considered in our work may be characterized along the taxonomy proposed in [12], as is characterized as follows:

- System network architecture: we adopt a centralized approach, with the goal of detecting attacks subverting the client-server model of CoAP in the range of the system detecting communications.
- Networking type: as is common in sensor network communication environments, a wireless hierarchical model is considered, with IEEE 802.15.4 at the link layer (supporting hop-by-hop communications), and 6LoWPAN, RPL and CoAP at the higher layers.
- Collection component: For traffic collection, we use an agent, in particular an IoT-LAB sniffer node capable of capturing the traffic in a specific location of the network.
- Data collection: data collection is centralized, given that the capture of the network traffic is performed in a specific node of the network.
- Data type: We store the captured wireless network traffic in the pcap (Packet Capture) format.
- Time of detection: in our implementation it is currently performed off-line.
- Granularity: in our current implementation we adopt a periodic (batch) approach to traffic capturing.
- Detection discipline: we consider the state-based and stimulating evaluation disciplines, since the IDS reports if a node is in the normal or compromised state. We identify the former as “NORMAL” throughout the article, and the latter as “INTRUSION”.
- Processing strategy: we adopt a centralized approach to processing the gathered data and to detect attacks.
- Detection methodology: as previously discussed, we currently consider, implement and evaluate anomaly-based CoAP intrusion detection in the context of the proposed framework.

We proceed by analyzing the proposed framework for intrusion detection and prevention in the context of Internet-integrated CoAP sensor networks.

### 3.2. System Architecture

**Figure 1:**
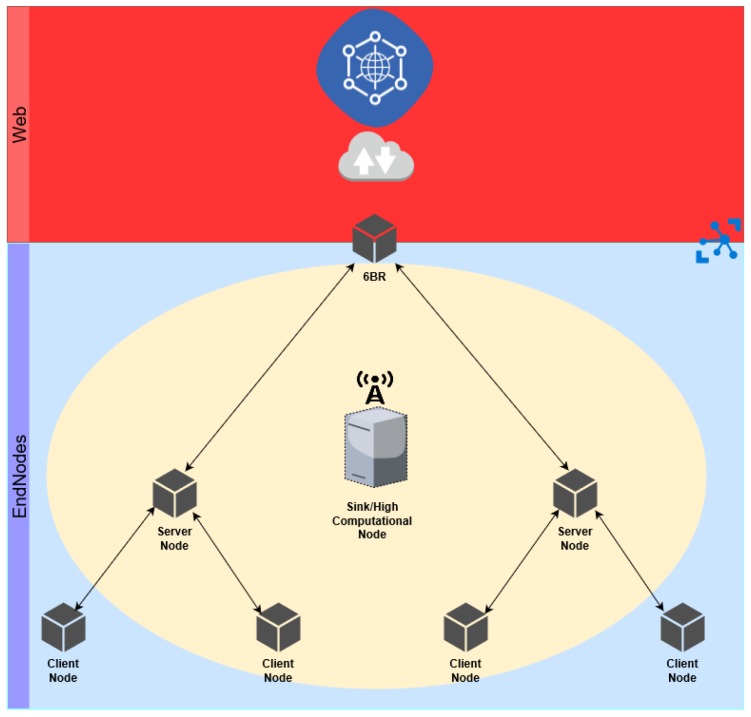
Architecture for intrusion detection in the context of Internet-integrated CoAP sensor networks.

We illustrate the architecture considered for the purpose of implementing and evaluating intrusion detection and prevention in Figure 1. This architecture considers the employment of a 6LoWPAN border router (6LBR) mediating communications between the WSN and Internet communications devices, the usage of CoAP sensing and actuating devices, and the clients of the resources supported by such devices, which may be either internal (in the same WSN domain) or external (in a different WSN domain or located in the Internet). Thus, CoAP clients associate with CoAP servers in order to request, actuate or observe particular resources available at the application-layer.

As illustrated in Figure 1, we assume the presence of a device employed to support intrusion detection operations. This device owns the resources required for performing capturing of the communications in its range, thus acting as a network sniffer. Another important requirement is that this device also supports a high level language interpreter in order to support the machine-learning algorithms employed for anomaly detection. In our current implementation of this architecture, this role is assumed by the 6LBR, and the fact that this device is responsible for filtering (mediating) the forwarding of communications between the Internet and WSN domains is also of help given that, after an intrusion has been detected, subsequent communications between the attacker and the attacked device may be denied. In the current implementation of the proposed architecture, CoAP server devices do not communicate with each other and CoAP client nodes request resources from servers at a rate controlled by a timer. As previously discussed, for the purpose of implementing and experimentally evaluating the considered architecture and the anomaly-detection mechanisms employed, we use the IoT-LAB [6] platform, configured to support a multi-hop topology. As a limitation of this platform, we consider requests to CoAP resources using GET requests only. Our implementation of the various devices in this architecture is performed in the Contiki operating system, in particular by building on top of the source code for the border-router (for the 6LBR), the er-example-server (CoAP server) and the er-example-client (CoAP client) implementations.

### 3.3. Misbehavior Detection

We find it important to start by identifying the threat model considered, and in this context we focus on internal attackers, thus devices that are able to participate in 6LoWPAN and CoAP communications [11], while at the same time trying to subvert the normal usage patterns, rules or semantics of the CoAP protocol. More precisely, we currently consider the detection of anomalies of four different (and complementary) classes of attacks against 6LoWPAN and CoAP communication environments and devices. We also label each class of attack, for the purpose of its employment with supervised machine learning algorithms, as follows:

- Label 1—Refers to CoAP requests sent to a CoAP server at a rate above a particular threshold, thus traducing an attack which we subsequently designate as “DoS FREQ”;
- Label 2—Refers to CoAP acknowledgements sent to a CoAP server when no corresponding CoAP requests exists, which we subsequently designate as “DoS ACK”;
- Label 3—Refers to requesting resources that are not supported (available) by the CoAP server, which we subsequently designate as “WRONG URI”;
- Label 4—Refers to sending requests to a CoAP server with an invalid ACCEPT option, which we subsequently designate as “WRONG ACCEPT”.

Other than the previous anomalous situations, the NORMAL class, using the label “0”, refers to the absence of an attack in the CoAP communications environment. The aforementioned situations are evaluated in the context of our framework and are known for not being detected by simple Signature-based IDS approaches. As such, this motivates our focus on the application of anomaly-based intrusion detection to CoAP communication environments. The presence of an attack reveals the compromised nodes, and as examples of anomalous behaviors we may consider an attacker trying to drain all the energy from the batteries of the compromised node, or forcing the remaining nodes to process unnecessary messages. Our discussion proceeds with an analysis of the machine learning methodology and of the algorithms employed for the purpose of implementing anomaly-based intrusion detection in the context of our framework.

### 3.4. Learning Methodology

Supervised Learning is a family of Machine Learning (ML) techniques that searches for the design of computational models capable of learning patterns from annotated data, in order to automatically classify new sets of unseen data. The performance of these techniques highly depend on the quality of the data and the parameters used in the configuration of the model, such as the kernel function and learning rates. These parameters must be selected carefully, in order to build models that can deliver good quality results.

Concerning the data used, it is preprocessed using Standardization, i.e., all of the features have a gaussian distribution of mean 0 and and standard deviation 1. This ensures that the features have the same underlying distribution, thus avoiding any misbehaviour from the ML algorithms. To extract features from the original data we employed the Principal Component Analysis (PCA) and Linear Discriminant Analysis (LDA).

The algorithm used to perform the detection of intrusions is the Support Vector Machines (SVM). SVMs are powerfull classification methods that perform well using reasonable amounts of computational resources. This is an important requirement to take into account on these systems, which makes the use of Neural Networks infeasible due to their excessive computational requirements and time-consuming learning process. Besides, SVMs have a faster classification procedure, which facilitates a real time implementation, and they have the advantage of being non-linear classifiers, based on the kernel function that is used.

**Figure 2:**
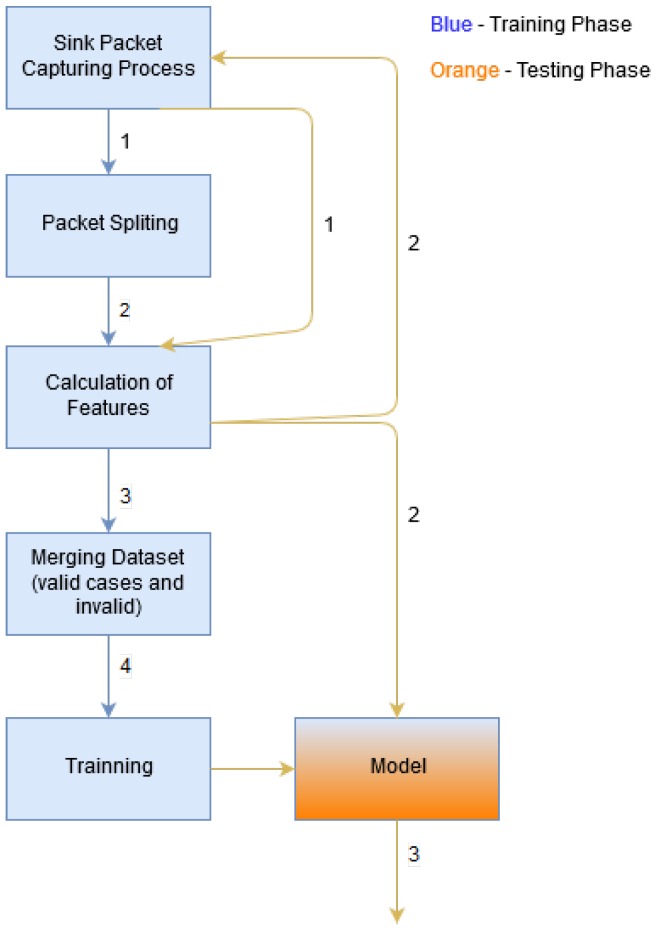
Approach considered for the Learning Methodology.

The intrusion detection mechanism was implemented using two different approaches: multi-class and binary-class. In the multi-class case we are interested in knowing which intrusion the system has detected. On the other hand, in the binary-class case we are simply detecting if there was an intrusion or not. To transform the multi-class problem into a binary-class one, we reduce the number of labels to two, where misbehavior labels were converted to a single label. For example, the multi-class array of labels [0; 1; 2; 1; 0; 3; 4; 2; 1] is converted into [0; 1; 1; 1; 0; 1; 1; 1; 1], where 0 means no intrusion and 1 means intrusion. The calculation of the features and the learning methodology considered are depicted in Figure 2. In this figure, the steps marked as “2” take place simultaneously, meaning that when the calculation of the features is completed the new data will be available for classification. Simultaneously, the network traffic starts to be obtained (using a network sniffer) again by the device supporting the IDS (the 6LBR in our implementation, as previously discussed in the context of Figure 1).

Referring again to Figure 2, in step “3” a set of labels is returned, and such labels are computed by applying the learned model to the test data. The process involves the splitting of packets and the calculation of features, and its aggregation is analyzed in greater detail in the context of the experimental evaluation, which we discuss next.

## 4. Experimental Evaluation

We proceed by analyzing the results obtained from our experimental evaluation of the proposed intrusion detection approaches, starting with an analysis on how the experiments were implemented and automated.

### 4.1. Implementation and Automation of the Experiments

The first step towards the implementation and automation of the experiments is the selection of an operating system to be employed in the constrained sensing devices, with the purpose of supporting the detection of attacks against the security of the network and devices. In addition, as our goal is to perform an overall experimental evaluation of the proposed mechanisms, it is necessary to select the platform to be employed for this purpose, as well as the tools to support the statistical data gatherer and the intrusion detection component in our architecture.

The operating system employed for the purpose of supporting intrusion detection is Contiki [5], due to its stability, hardware compatibility and the quality of the documentation at hand. As for the platform, we employed IoT-LAB [6], a platform which facilitates the process of deployment experiments, collecting and performing analysis on the results. With IoT-LAB, we are able to combine physical and simulation/emulation scenarios, and obtain real time data from any of the nodes in the network via Wireshark. IoT-LAB currently supports pressure, temperature, magnetometer, accelerometer, gyroscope and light sensors and, for the purpose of our experiments, we used M3 Open Nodes [28], due to the availability of network sniffers and power tracing tools using such devices.

**Figure 3:**
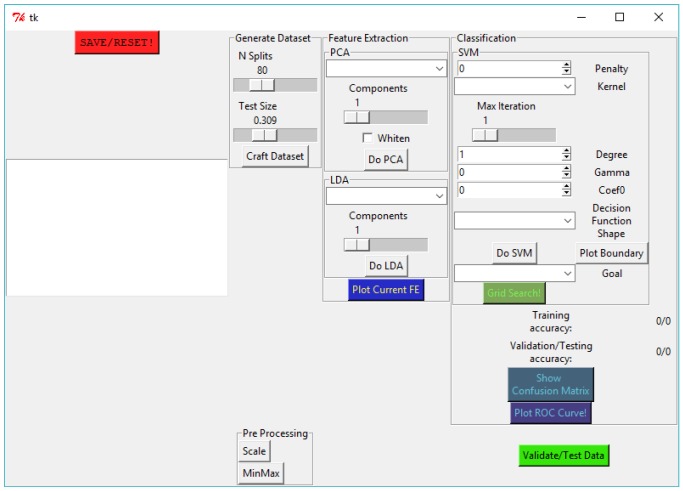
User interface developed to support the training phase.

**Figure 4:**
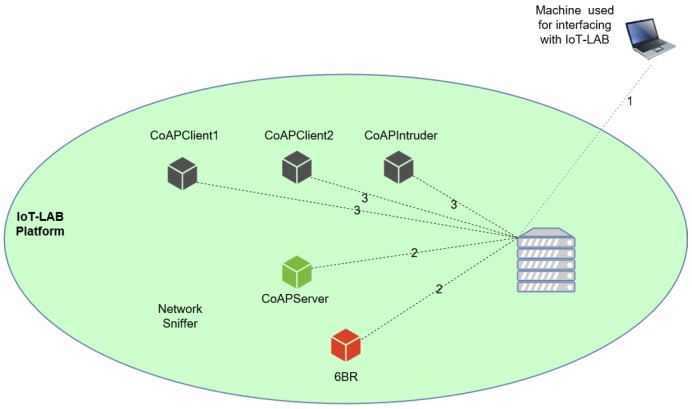
Methodology supporting the experimental measurements.

Regarding the intrusion detection component of our architecture, the ML application classifies nodes as either in the fully operational (normal) state or, on the other hand, as being compromised. Depending on the misbehavior detected for the node considered to be compromised, the node is labeled along the nomenclature discussed in Section 3.2. We developed an application to support the training phase of the IDS in Python 2, which also incorporates the GUI illustrated in Figure 3, developed in Tkinter. The library employed to support machine learning is Scikit-learn [29], and the plots were generated with matplotlib [30]. In our application, and by tuning the adequate parameters, we are able to choose to perform stratified splitting of the dataset into train/test parts with the desired proportion, and also perform pre-processing, feature extraction and classification. We have also implemented a functionality for performing a grid search for the best parameters of the classifier, according to the desired scoring goal. In Figure 4 we illustrate the methodology considered when building the experimental scenario employing the IoT-LAB platform.

The steps illustrated in Figure 4 reflect the various phases of the process employed to bootstrap the devices used in our experiments, to run the experiments and finally to retrieve information from the experimental measurements using the IoT-Lab platform. In Step 1 the local computer is connected to the IoT-Lab server in order to select the physical nodes to be employed in the experiments. In Step 2, the experiment is submitted to the platform, together with the specification of the firmware and a profile for the CoAP server and 6LBR devices. As for the CoAP client nodes, only a profile is associated, since the OS image of the client nodes is flashed after the IPv6 address of the CoAP server is known. In Step 3, we replace the hard-coded IPv6 address on the client nodes by the IPv6 address of the CoAP server, compiling the source code of the client and uploading the new image. After this last phase, the IoT-Lab setup is ready to support our experimental measurements.

In each experiment we employ a 6LBR, a CoAP server and three CoAP clients, among which one of the clients is employed to perform the attacks. We run each experiment during a period of 30 min, and a total of four experiments are performed with different attack (anomaly) scenarios. At the end of each experiment, the pcap capture file is analyzed by running Wireshark from Python, and employing the statistical capabilities of the network sniffer in order to calculate the features. Using Wireshark, we collect network data during a customized period of time, and perform an analysis at the end of that period. For the training phase, our approach was to split the entire training pcap file into subfiles, with the duration of the sampling period, using the editcap utility available with Wireshark.

### 4.2. Features Considered

In each experiment, a set of features are computed from the corresponding capture file, and we proceed by describing the features considered in our analysis.

#### 4.2.1. Features for Information from IEEE 802.15.4

- wpan−nonask−phy.frame_length—Frame length.
- wpan.aux_sec.frame_counter—Frame counter.
- wpan.bcn.gts.count—Guaranteed Time Slot (GTS) descriptor count.
- wpan.cmd.gts.length—GTS Length.
- wpan.correlation—Link Quality Indicator (LQI) correlation value.
- wpan.frame_length—Frame length.
- wpan.gtsreq.length—GTS length.
- wpan.sec_frame_counter—Frame counter.
- wpan.sec_key_sequence_counter—Key sequence counter.

#### 4.2.2. Features for Information from 6LoWPAN

- 6lowpan.frag.size—Datagram size.
- 6lowpan.fragment.count—Message fragment count.
- 6lowpan.hc2.udp.length—Length.
- 6lowpan.hops—Hop limit.
- 6lowpan.iphc.hlim—Hop limit.
- 6lowpan.mesh.hops—Hops left.
- 6lowpan.nhc.ext.length—Header length.
- 6lowpan.reassembled.length—Reassembled 6LoWPAN length.
- 6lowpan.udp.length—Length.

#### 4.2.3. Features for Information from IPv6

- ipv6.flow—Flow label.
- ipv6.fragment.count—Fragment count.
- ipv6.hlim—Hop limit.
- ipv6.opt.calipso.cmpt.length—Compartment length.
- ipv6.opt.jumbo—Payload length.
- ipv6.opt.length—Length.
- ipv6.opt.rpl.sender_rank—Sender rank.
- ipv6.plen—Payload length.
- ipv6.reassembled.length—Reassembled IPv6 length.
- ipv6.shim6.len—Length.
- ipv6.shim6.opt.elemlen—Element length.
- ipv6.shim6.opt.len—Length.
- ipv6.shim6.opt.total_len—Total length.

#### 4.2.4. Features for Information from CoAP

- coap.opt.block_size—Encoded block size.
- coap.opt.length—Options length.
- coap.opt.length_ext—Options extended length.
- coap.opt.max_age—Max-age.
- coap.token_len—Token length.
- coap.code—Status code.

As can be observed from the previous listing, the features identified belong in the context of the various communication protocols forming the standardized communications stack of the IoT [1], which we have previously discussed.

### 4.3. Evaluation Strategy

The classifiers of our intrusion detection approach are evaluated during the training phase, and in this context we consider fundamental scoring metrics of performance, in particular *Accuracy*, the *Recall (or True Positive Rate)*, *Precision*, *False Positive Rate* and *F_Measure*, defined as follows:

$$Recall=\frac{TP}{TP+FN}$$

$$Accuracy=\frac{TP+TN}{n}$$

$$Precision=\frac{TP}{TP+FP}$$

$$False\;Positive\;Rate=\frac{FP}{FP+TP}$$

$$\text{F\_Measure}=2\times \frac{Precision*Recall}{Precision+Recall}$$

In the previous definitions, *TP* represents the number of true positives, *TN* the number of true negatives, *FN* the number of false negatives, *FP* the number of false positives and *n* the number of observations considered. These performance metrics are evaluated considering confusion matrices and ROC (Receiving Operator Characteristic) curves, both in the multi and binary class approaches, which we proceed to analyze.

### 4.4. A Multi-Class Problem Approach

The results obtained from the analysis of the pcap files are constituted by observations, consisting of arrays of features organized as a rectangular matrix. Such observations are submitted to data pre-processing, dimensionality reduction and classifier training. The proportion of the number of NORMAL vs. erroneous observations (thus, resultant from the misbehaving node) is of 2 to 1, thus for each two NORMAL observations we observe one erroneous observation.

We split the classification problem into two problems. The first considers the four attacks and the normal behavior of the node, allowing a security manager to know the type of misbehavior present in the topology, if any. The second problem was approached from a binary problem perspective, and in this case the goal of the security manager is simply to detect the presence of an intrusion in the network. The two types of problems may yield different results, since the classifier will have in consideration that, for the binary class problem, the ERRONEOUS label is a combination of all the four labels of misbehavior. Moreover, the LDA feature extraction algorithm has in consideration the labels of the observations, which are different for the multi and the binary class problems.

**Figure 5:**
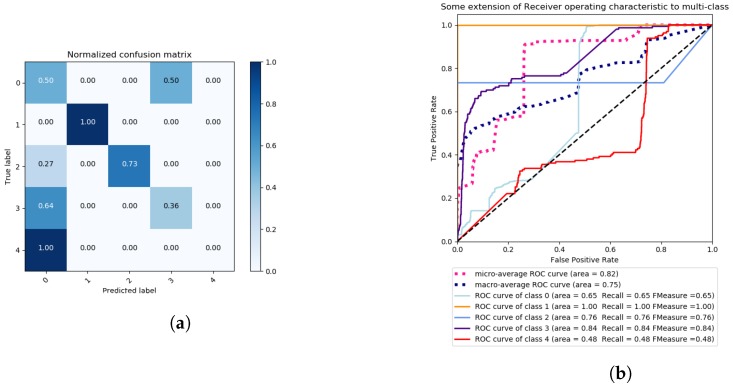
Multi-class problem—Grid Search SVM with: RBF Kernel, One vs. Rest decision function shape, 30 iterations and scoring criterion of accuracy, Confusion Matrix (**a**) and ROC Curves (**b**).

**Figure 6:**
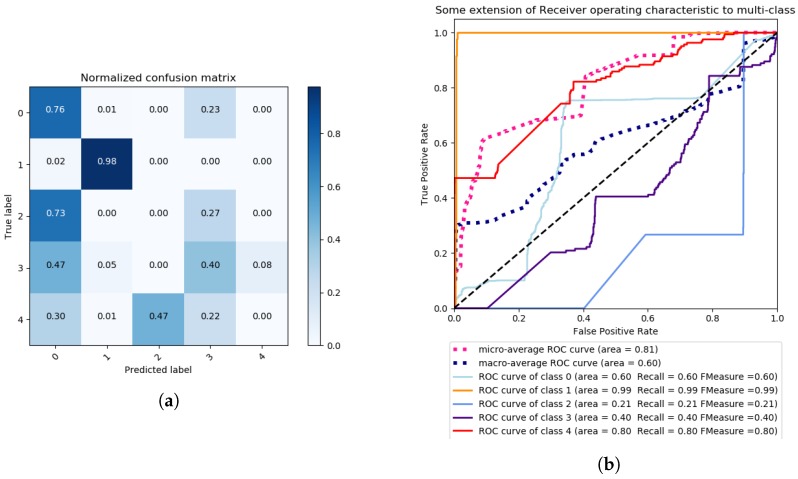
Multi-class problem—Grid Search SVM with: sigmoidal Kernel, One vs. Rest decision function shape, 30 iterations, and a scoring criterion of accuracy, confusion Matrix (**a**) and ROC Curves (**b**).

We employed two feature extraction algorithms, Principal component analysis (PCA) and Linear discriminant analysis (LDA). For both algorithms, we trained classifiers regarding the scoring metric of *Accuracy*. For PCA, the data is pre-processed with a Standard Scaler, mean 0 and standard deviation 1. The features of the data are reduced by PCA to 3 components with whitening, and the method is set to auto. Next, a grid search for the best parameters of the SVM classifier is performed, with a K-fold of 3 and setting the scoring metric to *Accuracy*. In this process we employed the following SVM Kernels: Linear, RBF, polynomial and sigmoidal. The obtained results are illustrated in Figure 5 and Figure 6.

For each considered Kernel, the best parameters and accuracy results are found by a grid search under the previously described conditions. The results obtained in this process are presented in Table 1.

As we can observe, PCA does not yield very good results while, nonetheless, RBF and sigmoidal kernels gave the best results on PCA in terms of accuracy, as shown in Table 1, with ∼51% for the RBF kernel and ∼62% for the sigmoidal kernel. Even with the previous two best kernels with PCA, there were cases of 0% of TP, being WRONG ACCEPT for the RBF kernel and DoS ACK for the sigmoidal kernel. According to the ROC curves of the previous best kernels, RBF has a higher recall and F_Measure on average than sigmoidal, scoring 76% for the DoS ACK attack, as illustrated in Figure 5b, versus the 21% of the same kernel, as in Figure 6b. The best result for sigmoidal kernel versus RBF kernel, in terms of F_measure and Recall, was with the WRONG ACCEPT attack, scoring 80%, versus the 48% of RBF kernel.

**Figure 7:**
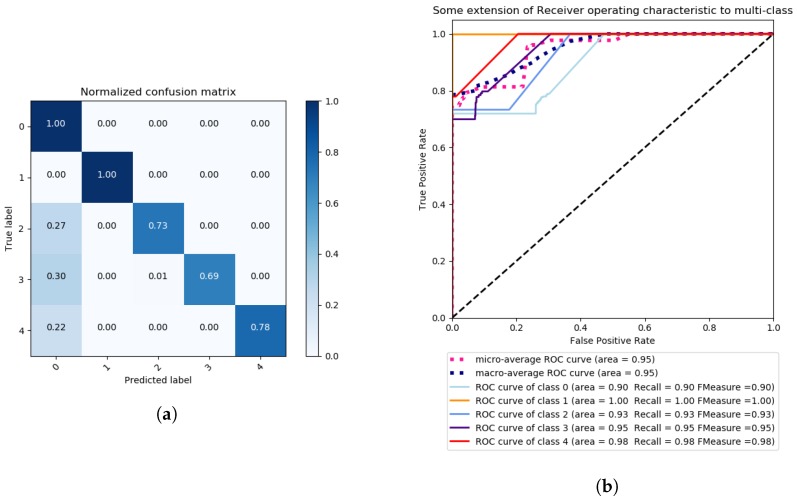
Multi-class problem—Grid Search SVM with polynomial Kernel, One vs. Rest decision function shape, 30 iterations, and scoring criterion of accuracy, Confusion Matrix (**a**) and ROC Curves (**b**).

**Figure 8:**
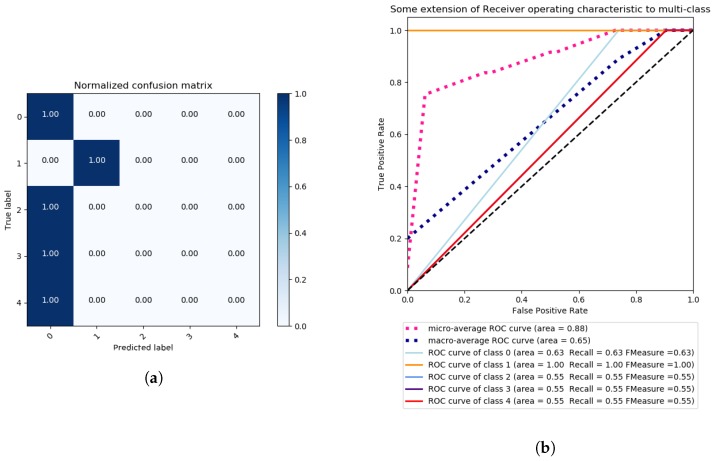
Multi-class problem—Grid Search SVM with sigmoidal Kernel, One vs. Rest decision function shape, 30 iterations, and a scoring criterion of accuracy, confusion Matrix (**a**) and ROC Curves (**b**).

In our next evaluation, LDA is used with the method of singular value decomposition and 6 components. Again, the data is pre-processed with a Standard Scaler, mean 0 and standard deviation 1. The results are illustrated in Figure 7 and Figure 8.

For each kernel, the best parameters and accuracy results are found by a grid search under the previously described conditions. The results obtained in this process are presented in Table 2.

As can be observed, the results show that LDA is better than PCA in terms of clustering the data according to each of the classes. By applying grid search with the same kernels, the polynomial kernel of degree 1 reaches a total accuracy of ∼93%, which corresponds to a linear kernel, versus the second best kernel, which is sigmoidal, with ∼75% of accuracy, as is visible in Table 2. In fact, sigmoidal kernel gave TPs of 0% for all attacks except DoS FREQ, with an almost 50% of average for the remaining attacks. ROC curves on the polynomial case also gave very high *Recall* and *F_Measure* scores, with the minimum of 90% for the DoS FREQ attack.

### 4.5. A Binary Class Problem Approach

**Figure 9:**
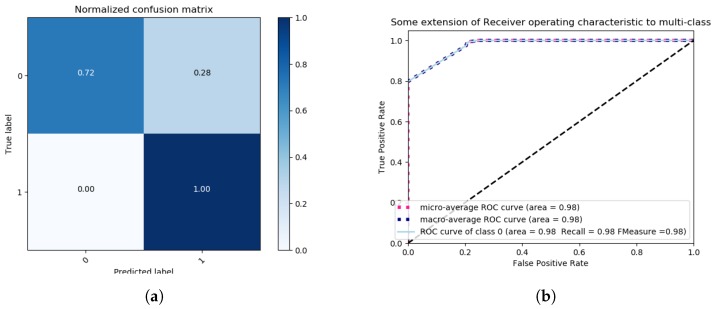
Binary class problem—Grid Search SVM with polynomial Kernel, One vs. Rest decision function shape, 30 iterations, with scoring criterion of accuracy, confusion Matrix (**a**) and ROC Curves (**b**).

**Figure 10:**
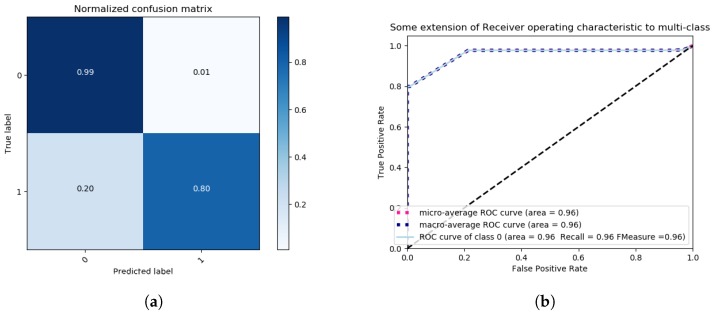
Binary class problem—Grid Search SVM with sigmoidal Kernel, One vs. Rest decision function shape, 30 iterations, and a scoring criterion of accuracy confusion matrix (**a**) and ROC Curves (**b**).

We proceed by discussing the results obtained by following the binary class problem approach. For this approach, we considered the following two labels in our evaluation: NORMAL with Label “0” and ERRONEOUS with Label “1”. For this approach, we only employ the most accurate feature extraction method, based on the previous obtained multi-class results, and that is LDA. The method of singular value decomposition and two components is applied. Once more, data is pre-processed with a Standard Scaler, with mean 0 and standard deviation 1. The features of the data are reduced by LDA to 2 components, and the method is set to singular value decomposition (SVD). Next, a grid search for the best parameters of the SVM classier is performed, with a K-fold of 3 and setting the scoring metric to *Accuracy*. We employ the following SVM Kernels: Linear, RBF, polynomial and sigmoidal, and the obtained results are illustrated in Figure 9 and Figure 10.

For each Kernel, the best parameters and accuracy results are found by a grid search under the previously described conditions. The corresponding results are presented in Table 3. As can be observed, the Sigmoidal Kernel is able to accurately classify NORMAL observations with a ratio of false positives of 1%, as can be seen in Figure 10a, though with an amount of false negatives of 20%. For the polynomial Kernel, there are 0% false negatives and 28% of false positives, as can be observed in Figure 9a. In terms of recall, both polynomial and sigmoidal kernels present similar results, polynomial being 2% ahead of sigmoidal, as can be seen in Figure 9b and Figure 10b.

In conclusion, from our previous discussion we may note that, in our work, we employ pre-processing algorithms and train classifiers for building the intrusion model, according to the multi and binary class approaches. As we have observed from the results of our experimental evaluation, for the multi-class problem approach the proposed system is capable of distinguishing between the four types of misbehaving scenarios implemented, and to reach an accuracy of 93% for the best SVM classier, with accuracy as the scoring metric and when employing LDA to perform feature extraction. As for the binary class problem approach, considering the best SVM parameters, a classification of NORMAL and ERRONEOUS behaviors can reach an accuracy of 92% and an *F_Measure* of 98%.

## 5. Conclusions and Future Work

In this article we have proposed an IDS framework for the detection and prevention of attacks in Internet-integrated CoAP networks, and in the context of this framework we have evaluated the effectiveness of employing anomaly-based intrusion detection in preventing DoS attacks against such communication environments. From a practical perspective of implementing an IDS system in Internet-integrated CoAP sensing applications, it is crucial to prevent the most intrusions as possible, while at the same time assuring a low rate of false negatives, even if at a cost of increasing false positives. This must be taken into account when searching for the maximum accuracy, and from our results we may also consider that, in the case that the security manager is interested and able to identify particular attacks, the multi-class problem approach is appropriate, since results show that a linear Kernel or a polynomial Kernel have the best results regarding accuracy. On the other hand, the binary class approach is a good fit to capture the largest possible amount of anomalous (ERRONEOUS) behaviors. In general, we consider that the results obtained from our experimental evaluation show that anomaly-based intrusion detection is viable to protect 6LoWPAN and CoAP communication environments from internal and Internet-originated attacks against the security and stability of the devices.

Although only SVM was used as a classier algorithm, the proposed system can be extended to include additional algorithms, such as K-NN, Neural Networks or Random Forests. Thus, the implementation and evaluation of such alternative algorithms in the context of the proposed framework is part of our plans to conduct further research work in this area. The inclusion of other algorithms will also provide an opportunity to evaluate the scalability of the proposed system using machine learning models that more demanding in terms of computational resources such as delay, storage and computational effort. In addition, and from a more practical implementation standpoint, another aspect that we plan to target is related to how the features are currently calculated. The computational efficiency in this step can certainly be improved by developing an application for calculating features directly from the captured data. Additional intrusions will also be considered in future developments, particularly regarding attacks focused on subverting the usage rules and the semantics of the CoAP protocol, either by internal or external attackers.

## Figures and Tables

**Table 1:** SVM Parameters obtained using accuracy as the scoring metric (previously processed with multi-class PCA).

Scoring Metric: accuracy							
C	Kernel	Iterations	Degree (Valid on Polynomial only)	Gamma	Coef0	Decision Function Shape	Accuracy
0.957895	‘linear’	30	1	0.100000	0.000000	‘ovr’	0.212513
0.957895	‘rbf’	30	1	1.000000	0.000000	‘ovr’	0.509824
0.410526	‘poly’	30	2	0.621053	0.105263	‘ovr’	0.283868
0.200000	‘sigmoid’	30	1	0.194736	0.947368	‘ovr’	0.618408

**Table 2:** SVM Parameters obtained using accuracy as the scoring metric (previously processed with multi-class LDA).

Scoring Metric: accuracy							
C	Kernel	Iterations	Degree (valid on Polynomial only)	Gamma	Coef0	Decision Function Shape	Accuracy
0.452632	‘linear’	30	1	0.100000	0.000000	‘ovr’	0.932782
0.578947	‘rbf’	30	1	0.810526	0.000000	‘ovr’	0.632368
0.200000	‘poly’	30	1	0.147368	0.000000	‘ovr’	0.933816
0.284211	‘sigmoid’	30	1	0.100000	0.000000	‘ovr’	0.753361

**Table 3:** SVM Parameters obtained using accuracy as the scoring metric (previously processed with dual-class LDA).

Scoring Metric: accuracy							
C	Kernel	Iterations	Degree (valid on Polynomial only)	Gamma	Coef0	Decision Function Shape	Accuracy
0.200000	‘linear’	30	1	0.100000	0.000000	‘ovr’	0.858325
0.200000	‘rbf’	30	1	0.100000	0.000000	‘ovr’	0.830403
0.915790	‘poly’	30	3	0.621053	0.000000	‘ovr’	0.810238
0.200000	‘sigmoid’	30	1	0.9052632	0.315710	‘ovr’	0.927094

## References

[1] Palattella M.R., Accettura N., Vilajosana X., Watteyne T., Grieco L.A., Boggia G., Dohler M. (2013). Standardized protocol stack for the internet of (important) things. IEEE Commun. Surv. Tutor..

[2] Montenegro G., Kushalnagar N., Hui J., Culler D. Transmission of IPv6 Packets Over IEEE 802.15.4 Networks. https://tools.ietf.org/html/rfc4944.

[3] Winter T., Thubert P., Brandt A., Hui J., Kelsey R., Levis P., Pister K., Struik R., Vasseur J.P., Alexander R. RPL: IPv6 Routing Protocol for Low-Power and Lossy Networks. http://www.rfc-editor.org/info/rfc6550.

[4] Bormann C., Castellani A.P., Shelby Z. (2012). Coap: An application protocol for billions of tiny internet nodes. IEEE Int. Comput..

[5] Dunkels A., Gronvall B., Voigt T. Contiki-a lightweight and flexible operating system for tiny networked sensors. Proceedings of the 29th Annual IEEE International Conference on Local Computer Networks.

[6] FIT IOT-LAB Iot Experimentation at a Large Scale. https://www.iot-lab.info/.

[7] Howitt I., Gutierrez J.A. IEEE 802.15.4 low rate—wireless personal area network coexistence issues. Proceedings of the 2003 IEEE Wireless Communications and Networking (WCNC 2003).

[8] Nieminen J., Savolainen T., Isomaki M., Patil B., Shelby Z., Gomez C. Ipv6 Over Bluetooth(r) Low Energy. http://www.rfc-editor.org/info/rfc7668.

[9] Granjal J., Monteiro E., Silva J.S. (2015). Security for the internet of things: A survey of existing protocols and open research issues. IEEE Commun. Surv. Tutor..

[10] Le A., Loo J., Lasebae A., Aiash M., Luo Y. (2012). 6LoWPAN: A study on QoS security threats and countermeasures using intrusion detection system approach. Int. J. Commun. Syst..

[11] Shelby Z., Hartke K., Bormann C. The Constrained Application Protocol (CoAP). http://www.rfc-editor.org/info/rfc7252.

[12] Liao H.J., Lin C.H.R., Lin Y.C., Tung K.Y. (2013). Intrusion detection system: A comprehensive review. J. Netw. Comput. Appl..

[13] Can O., Sahingoz O.K. A survey of intrusion detection systems in wireless sensor networks. Proceedings of the 2015 6th International Conference on Modeling, Simulation, and Applied Optimization (ICMSAO).

[14] Aljawarneh S., Aldwairi M., Yassein M.B. (2018). Anomaly-based intrusion detection system through feature selection analysis and building hybrid efficient model. J. Comput. Sci..

[15] Gai K., Qiu M., Tao L., Zhu Y. (2016). Intrusion detection techniques for mobile cloud computing in heterogeneous 5G. Secur. Commun. Netw..

[16] Kasinathan P., Pastrone C., Spirito M.A., Vinkovits M. Denial-of-Service detection in 6LoWPAN based Internet of Things. Proceedings of the 2013 IEEE 9th International Conference on Wireless and Mobile Computing, Networking and Communications (WiMob).

[17] Oliveira L.M.L., Rodrigues J.J.P.C., de Sousa A.F., Lloret J. (2013). Denial of Service Mitigation Approach for IPv6-enabled Smart Object Networks. Concurr. Comput. Pract. Exp..

[18] Suricata Home Page. https://suricata-ids.org/.

[19] Raza S., Wallgren L., Voigt T. (2013). SVELTE: Real-time intrusion detection in the Internet of Things. Ad Hoc Netw..

[20] Sicari S., Rizzardi A., Miorandi D., Coen-Porisini A. (2018). REATO: REActing to Denial of Service attacks in the Internet of Things. Comput. Netw..

[21] Sellami L., Idoughi D., Baadache A. (2014). Intrusions Detection System Based on Ubiquitous Network Nodes. arXiv.

[22] Roesch M. (1999). Snort: Lightweight intrusion detection for networks. Lisa.

[23] Cho E.J., Kim J.H., Hong C.S., Hong C.S., Tonouchi T., Ma Y., Chao C.S. (2009). Attack Model and Detection Scheme for Botnet on 6LoWPAN. Management Enabling the Future Internet for Changing Business and New Computing Services.

[24] Gupta A., Pandey O.J., Shukla M., Dadhich A., Mathur S., Ingle A. Computational intelligence based intrusion detection systems for wireless communication and pervasive computing networks. Proceedings of the 2013 IEEE International Conference on Computational Intelligence and Computing Research.

[25] Pongle P., Chavan G. (2015). Real time intrusion and wormhole attack detection in internet of things. Int. J. Comput. Appl..

[26] Summerville D.H., Zach K.M., Chen Y. Ultra-lightweight deep packet anomaly detection for Internet of Things devices. Proceedings of the 2015 IEEE 34th International Performance Computing and Communications Conference (IPCCC).

[27] Saeed A., Ahmadinia A., Javed A., Larijani H. (2016). Intelligent Intrusion Detection in Low-Power IoTs. ACM Trans. Int. Technol..

[28] FIT IOT-LAB M3 Open Node. https://www.iot-lab.info/hardware/m3/.

[29] Pedregosa F., Varoquaux G., Gramfort A., Michel V., Thirion B., Grisel O., Blondel M., Prettenhofer P., Weiss R., Dubourg V. (2011). Scikit-learn: Machine learning in Python. J. Mach. Learn. Res..

[30] Hunter J.D. (2007). Matplotlib: A 2D graphics environment. Comput. Sci. Eng..

